# The Relationship Between Orthodontic Abnormalities and Spinal Deformities: A Review of Literature

**DOI:** 10.7759/cureus.80934

**Published:** 2025-03-21

**Authors:** Ylenia Pastorello, Patrik Buzgau, Ioana Theodora Barna, Lóránd Dénes

**Affiliations:** 1 Department of Anatomy and Embryology, George Emil Palade University of Medicine, Pharmacy, Science and Technology of Târgu Mureș, Târgu Mureș, ROU; 2 Faculty of Medicine, George Emil Palade University of Medicine, Pharmacy, Science and Technology of Târgu Mureș, Târgu Mureş, ROU; 3 Faculty of Dentistry, George Emil Palade University of Medicine, Pharmacy, Science and Technology of Târgu Mureș, Târgu Mureş, ROU

**Keywords:** malocclusion, posture, spinal abnormalities, stomatognathic system, temporomandibular joint disorders

## Abstract

Spinal deformities comprise abnormal curvatures of the spine which impact posture, musculoskeletal balance, as well as biomechanical function. Malocclusion, the improper contact between occlusal surfaces of opposing teeth, affects the occlusal relationship and functionality of the temporomandibular joint (TMJ). While both conditions have been extensively studied independently, their interconnection remains a subject of debate. The aim of this work is to comprehensively review the current literature concerning spinal curvature abnormalities and stomatognathic alterations, concomitantly investigating the relationship between these disorders.

Research findings highlighted how changes in the TMJ biomechanics may modify occlusion patterns affecting head posture and, consequentially, spinal alignment. Proprioceptive feedback from the periodontal ligament and trigeminal nerve plays a crucial role in controlling muscle tension and, therefore, normal TMJ function. Disruptions in this feedback contribute to occlusal alterations which, in turn, may induce postural adaptations influencing the spinal curvature. Studies also demonstrated a statistical correlation between adolescent idiopathic scoliosis (AIS) and orthodontic anomalies such as overbite, overjet, and mandibular retrusion. Additionally, reverse chewing cycles (RCCs), a characteristic of crossbite, have been found to be more prevalent in individuals with scoliosis, suggesting a possible link between dysfunctional mastication and spinal deformities. Considering the complex interplay between occlusion and posture, a multidisciplinary approach to diagnosis and treatment is regarded as essential. While orthodontic treatment effectively corrects malocclusion, evidence suggests that dental bracing may influence TMJ function and spinal alignment, especially in pediatric patients still subject to growth spurts. Despite emerging data indicating a relationship between malocclusion and spinal deformities, further research is required in order to clarify such causality and promote the creation of therapeutic schemes for patients concurrently affected by these pathologies.

## Introduction and background

The spine represents an integral part of the human endoskeleton, consisting of 33-34 stacked vertebral bodies altogether delimiting the vertebral canal which houses the spinal cord. While the distal part of the spine is fused together, lumbar, thoracic and cervical vertebral bodies allow for a restricted range of motion through facet joints and moderate shock absorption via intervertebral discs [1].

The stomatognathic system is responsible for mastication, ingestion of bolus, word formation and articulated speech. Its functionality depends on the anatomical relationship between various craniofacial structures, such as the mandible, maxilla, teeth and the temporomandibular joint (TMJ) [2].

A sizable proportion of patients diagnosed with spinal deformities also present with associated occlusal patterns or TMJ disorders (TMD) [3,4]. This finding suggests that a link may be present between the two coexisting diagnoses. While both are well-researched independently, their association is largely unexplored and even divisive in current literature [5]. This narrative review aims to provide a state-of-the-art description of spinal curvature abnormalities and stomatognathic alterations, concurrently exploring the relationship between these pathologies.

Methodology

An extensive search was conducted using international literature databases comprising PubMed, ScienceDirect, Google Scholar, MEDLINE, and the Cochrane Library. Articles containing the keywords “spinal abnormalities”, “posture”, “stomatognathic system”, “temporomandibular joint disorders”, and “malocclusion” were included.

## Review

Spinal deformities: kyphosis, lordosis, and scoliosis

Adult spinal deformities (ASDs) include a heterogeneous group of lumbar or thoracolumbar idiopathic or degenerative conditions implicating deviations of the vertebral column in the coronal or the sagittal plane [6]. Kyphosis represents a posteriorly convex or outward sagittal deviation of the spine. Postural kyphosis is related to improper posture maintained, a complication of tuberculosis, or can be associated with Scheuermann's disease [7-9].

Lordosis consists of an inward or posteriorly concave deviation of the lumbar part of the vertebral column within the sagittal plane. For a long time, the lordotic curvature of the spine, particularly at the transition with the sacrum, has been noted as an evolutionary weak point, as it has been connected with several degenerative conditions [10]. This association is, however, controversial as spinal degenerative conditions of similar rates have been found to be developing in primates without a naturally occurring lordotic curvature [11]. The female spine has a greater curvature with a caudally located lordotic peak and increased cranial height. While caudal height is similar between males and females, the impression of lordosis is usually accentuated in females due to the superior part of their lumbar curvature being more posteriorly inclined [12]. It is important to mention that a lordotic curvature presents a series of mechanical advantages, as it is largely considered to be a human adaptation developed over time to aid in posture maintenance and gait [13].

Scoliosis represents a coronal deviation of the spine resembling an S or C shape. While most reported cases are asymptomatic and do not require treatment, serious cases are life-altering and can require surgical correction. Classification includes idiopathic, congenital, or neuromuscular types. Idiopathic scoliosis is subdivided into infantile, juvenile, and adolescent categories [14].

Adolescent idiopathic scoliosis (AIS) is diagnosed in children between 10 and 18 years old, representing the most common form of pediatric scoliosis and accounting for up to 90% of diagnoses [15]. Neuromuscular scoliosis is associated with improper muscular stabilization of the spine such as spinal muscular atrophy or cerebral palsy and other muscle dystrophies [16].

Congenital scoliosis represents a spinal deformity occurring due to failure of normal vertebral development and/or segmentation, leading to an anomalous curvature of the spine. This pathology is present at birth but the patient might not display signs and symptoms until puberty [17]. While it has been associated with polygenic defects, its etiology is still unclear [18].

Clinical presentations for patients with spinal deformities include general symptoms applicable to all subtypes discussed previously. The similarities include lower back pain (LBP) or discogenic pain, neurogenic claudication caused by moderate to severe stenosis of the spinal canal, or exiting neural foramina [19,20]. It is common for patients experiencing pain associated with spinal stenosis to not obtain relief upon changing positions and to also present with radicular leg pain or weakness [21].

Spinal deformities are diagnosed via radiological imaging necessary for general evaluation and calculating specific parameters. The most useful parameter used especially in diagnosing scoliosis is the Cobb angle. The main Cobb angle is measured by identifying the largest curve and its two end vertebrae. This method is then utilized by drawing lines along the superior border of the cranial and the inferior border of the caudal end vertebrae to form the Cobb angle [22]. For a scoliosis diagnosis, the angle’s value must exceed 10°, with severe cases exceeding 45°. Severe scoliosis cases are associated with reduced space in the thoracic cavity and result in improper respiratory function [23]. Cobb angle measurements are redone over time to measure the disease's severity or progression, especially in AIS cases, where the spine is still growing [24].

Stomatognathic system and its alterations

The TMJ is a synovial joint between the mandibular fossa located on the temporal bone and the condylar process of the mandible. Uncommonly, the articular surface is lined with fibrocartilaginous tissue rather than hyaline cartilage, exchanging unrestricted mobility for increased shock absorption and buffering capacity [2]. The joint allows for both translational and rotational movements; however, its movement range does not have a precise endpoint. The terminal position of the condylar process is highly dependent on how occlusion is achieved [25].

Dental occlusion is the contact realized between the occlusal surface of opposing teeth from both the superior and inferior arch. Orthodontic anomalies, such as overbite, overjet, or absence of teeth (anodontia), can have significant implications on how occlusion is achieved and therefore influences TMJ function overall [26].

Mastication represents a sequence of rhythmic mandibular movements allowing for food manipulation and initial digestion of ingested bolus. It is the result of a collective effort between the masseter, temporalis, suprahyoid, facial muscles, and the tongue, directing food to and from the teeth [27]. While many craniofacial structures are involved, the TMJ is responsible for most of the efficiency and function of mastication. Neuromuscular control of the TMJ involves the inferior alveolar, maxillary, and infraorbital nerves as well as the auriculotemporal nerve that extends to the surface of the articular disc involved in the joint [28,29]. The trigeminal nerve is the fifth cranial nerve, consisting of three main divisions that exit through the Meckel cavity in the middle cranial fossa from the trigeminal ganglion. The mandibular nerve, responsible for motor control of masticatory muscles, exits into the infratemporal fossa through the foramen ovale and divides into multiple branches, one of which is the inferior alveolar nerve (IAN) [30]. After emerging from the mandibular nerve, the IAN enters the mandibular canal where it emits sensory branches resulting in the inferior dental plexus. It terminates distally with the mental nerve and the incisive branch. The superior dental plexus is created from the anterior superior alveolar nerve (ASAN), middle superior alveolar nerve (MSAN), and the posterior superior alveolar nerve (PSAN), all branches originating from the infraorbital division of the maxillary nerve [31].

Sensory information from the teeth is received via mechanoreceptors in the periodontal ligament (PDL). The PDL represents the link between the mineralized surfaces of the dental root and alveolar bone. It functions as a shock absorber but also as an important component in achieving neuromuscular control of the TMJ in order to regulate muscular contraction forces in mastication [32]. Mechanoreceptors in the PDL transmit information about food density and texture but also sensitive feedback upon contact with the opposing tooth's occlusal surface. The trigeminal loop refers to a pattern of motion for the mandibular excursion and progressively mediated tension during contraction of the masseter and temporalis muscles, relying on continuous sensory feedback from the mechanoreceptors in the PDL by way of the mesencephalic nucleus of the trigeminal nerve [33].

Malocclusion

Malocclusion, as regarded by the WHO, represents the third most prevalent oral health issue after caries and periodontal disease [34]. It is not considered a disease but a deviation from normal occlusion, occurring because of genetic and environmental factors [35]. It is defined as the improper contact between occlusal surfaces of opposing teeth and can develop after the emergence of mixed dentition. In Ackerman and Profitt's classification system, malocclusion is divided into classes I, II, III, IV, V, and VI. These categories are based on the relationship between the maxillary and mandibular teeth and the jaw position.

Class I: The maxillary teeth are slightly forward of the mandibular teeth, and the jaw is aligned properly.

Class II: The maxillary teeth are significantly forward of the mandibular teeth, and the jaw is underdeveloped.

Class III: The mandibular teeth are significantly forward of the maxillary teeth, and the jaw is overdeveloped.

Class IV: The maxillary teeth are significantly behind the mandibular teeth.

Class V: The maxillary teeth are significantly forward of the mandibular teeth, and the jaw is overdeveloped.

Class VI: The mandibular teeth are significantly behind the maxillary teeth [36,37].

Malocclusion can be diagnosed by a primary care dental provider by performing a basic oral examination. Further investigations can be made using extraoral imaging studies such as panoramic x-rays or cone beam computed tomography (CBCT) [38].

Treatment plans are managed by an orthodontist and include palatal expanders, spacers, and braces. Palatal expanders work by adding pressure on the maxillary bones to split the mid-palatal suture and expand the volume of the oral cavity. They are especially useful in cases of overcrowding, narrow palate, or overbite. Treatment is commenced typically before other orthodontic procedures, such as bracing, and is recommended for adolescents as the maxillary bone completely fuses post-puberty. Bracing represents the most widely used and available orthodontic treatment, consisting of placing metal brackets on the dental surfaces and connecting them using a metal archwire that is progressively tightened over time. While highly effective, bracing can expose the patient to long-term gingivitis and negatively impact daily activities such as mastication and speaking. Spacers increase interproximal space between teeth and can be used alongside braces to allow for optimal bracket placement [39].

Relationship between malocclusion and TMD

Proprioception plays a significant role in TMJ function and achieving normal occlusion. While anterior teeth exhibit more sensitivity, the canines contain the largest number of proprioceptors and are the last permanent teeth to fall [40].

The PDL represents a structural connection between the tooth and its alveolar socket [41]. With a high density of mechanoreceptors (Ruffini endings) also capable of nociception, it collectively relays information to the trigeminal nuclei through the superior and inferior dental plexus [42]. Sensory feedback allows for a reduction of occlusal forces, however, altered feedback caused by disharmonic occlusion can influence chewing patterns by increasing muscle tension [43].

Contact of the upper cuspids during mastication triggers sensory feedback especially from the canines, signaling the fifth cranial nerve and protecting the TMJ. This anterior guidance also enables the temporal and masseter muscles to relax and further prevents clenching [44,45].

Physiologically, the interaction between the jaw condyle and the squamous part of the temporal bone is limited by the normal occlusion of both maxillary and mandibular teeth. Abnormal occlusal patterns thus influence the anatomical position of the condyle and over time, influence the myoelectric activity of the craniofacial muscles and masticatory patterns [46]. By altering occlusal patterns with cotton rolls inserted for bilateral and subsequent unilateral support, and reviewing electromyographic (EMG) data from both the temporalis and masseter muscles during voluntary clenching in the intercuspal position, a study performed on 47 healthy individuals demonstrated a range of variability in EMG activity when occlusal patterns were altered [47].

A study conducted by Aboalnaga et al. assessed the dental and skeletal aspects of malocclusion, in the anteroposterior and vertical dimensions, in 150 patients previously diagnosed with TMD and correlated such aspects with the signs and symptoms of TMD. Although the relations between occlusal variables and TMD parameters proved to be insignificant in the myogenic TMD group, statistical significance was reached between centric slides and total muscle pain score in the joint disorders group [48]. Furthermore, a meta-analysis comparing the prevalence of TMDs in patients with malocclusion to subjects presenting normocclusion concluded that TMDs and related symptoms are more commonly associated with malocclusion class II, III, anterior open or deep bite, and increased overjet or overbite, therefore confirming the adverse effects of malocclusion on the masticatory system [49].

Relationship between the TMJ and posture

As previously discussed, the existence of occlusal patterns does interfere with normal TMJ function and mandible position. The following analysis aims to assess the possible connection between mandible position/TMJ function and body posture, therefore verifying if postural deficits, occlusal patterns and TMDs are related in etiology and development.

The craniocervical junction (CCJ) represents the connection between the head and neck, providing vital biomechanical functionality and stability. It is composed of the atlantooccipital joint as well as the atlantoaxial segment [50].

A deviation in the balance of the CCJ would not allow for the head to maintain a neutral position, therefore creating an imbalance of forces that would be transmitted to the TMJ, via the temporal bone. This deviation would, in return, have the potential of altering proper occlusion [25].

Multiple studies have assessed the relationship between jaw movement and body position with initial analysis going as far back as 1926. While investigating children with airway obstruction, Schwartz concluded that increased craniocervical angles and forward cervical inclination were associated with abnormal breathing patterns [51]. Further research has also associated a tilted head posture and vertical facial development with malocclusion. Participants were asked to perform a series of standardized balance tests further concluding that different classes of malocclusions predicted a forward or backward postural tilt [52].

The purpose of a cephalometric analysis is to evaluate orthodontic treatment options for patients when considering anteroposterior movement as part of the therapeutic strategy. Cephalometric analysis evaluates the anteroposterior and vertical relationships of the mandible and maxilla with the cranial base and each other, and the relationships of the upper and lower teeth to the mandibular and maxillary bones. Because of anatomical variability, comparing an individual patient’s measurements to a standard may not always be conclusive, thus all values need to be interpreted in the clinical context [53]. The sella-nasion-B point (SNB) angle, measures the position of the mandible relative to the position of the cranial base. It is measured indeed between the SNB points (the most concave point on the mandibular symphysis) with a mean value of 78°±3 [53,54]. In a study evaluating 94 patients with postural deformities, Šidlauskienė et al. concluded that there is a correlation between kyphotic posture and increased SNB angle and that patients with kyphosis also associated naso-laryngeal obstruction [55].

The De Giorgi study (2018) assessed the effect of an occlusal splint on the posture of intra-articular TMD patients. The evaluated postural parameters included trunk inclination, cervical and lumbar arrows, kyphotic and lordotic angles, trunk imbalance, pelvic tilt, and torsion, which were analyzed at baseline, and after one, three, and six months by rasterstereographic recordings. A statistically significant difference was reached between the intervention and the control group for the cervical arrow at rest position (horizontal distance between vertebra prominens and the tangent to the curve at kyphosis apex parallel to the vertebra prominens-midpoint between lumbar dimples axis) at a one-month check-up, as well as the kyphotic angle between the cervicothoracic inflection point and the thoracolumbar inflection point at rest position (at one- and three-month assessment). Concerning the lordotic angle between the thoracolumbar inflection point and the lumbosacral inflection point, a statistically significant difference was reported at rest position at the three-month evaluation [56].

Malocclusion and spinal deformities

AIS has been often associated, although somewhat controversially in the past, with malocclusion. A recent study evaluated 14-16-year-old patients diagnosed with AIS by taking alginate impressions in the maximal intercuspal position. By comparing their findings with a control group, a significant association between AIS and malocclusion traits such as overbite or overjet was found [57]. Laskowska et al. evaluated 80 children and adolescents diagnosed with scoliosis next to a control group and concluded that there is a significant statistical correlation between children diagnosed with AIS and the presence of at least one malocclusion trait [58]. A study conducted by Saccomanno et al. on 477 scoliotic patients reported a significant correlation between the presence of scoliosis and malocclusion, with the pre-existence of these conditions within the family being the best predictor [59].

Reverse chewing cycles (RCCs) represent dyskinetic masticatory patterns associated with the development of crossbite [60]. Piancino et al. aimed at investigating the relationship between AIS and alteration of chewing patterns in patients specifically without a preexisting crossbite diagnosis, to exclude RCCs as a result of the malocclusion. Motion was recorded with the head in a fixed position, alternating between chewing on one side or the other with soft and hard bolus. Results showed a significant statistical relationship between RCCs and AIS, therefore demonstrating a link between alternative chewing patterns and spinal deformities [61].

**Figure 1:**
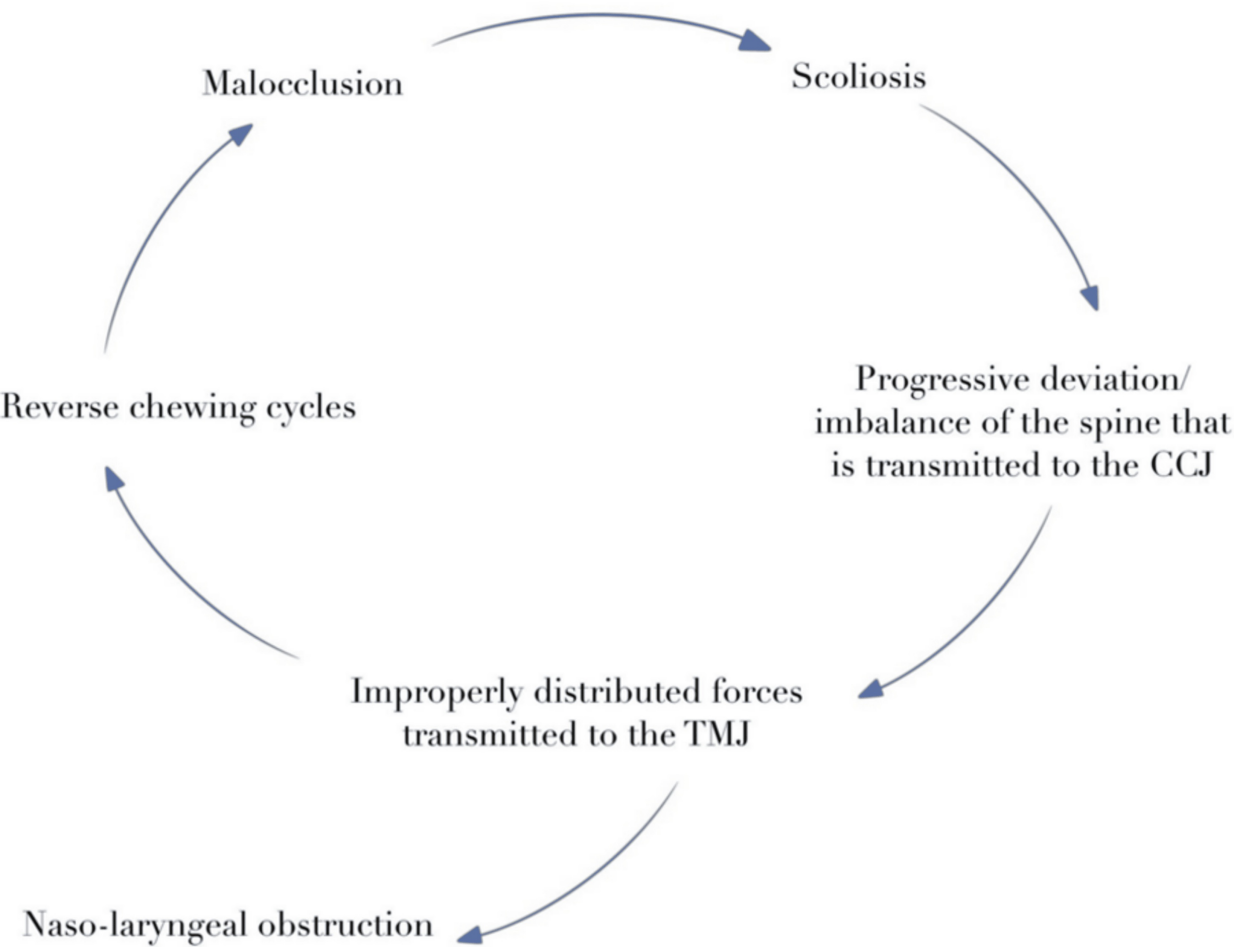
Relationship between scoliosis, postural deficits, and malocclusion. Spinal deformities, such as scoliosis, are characterized by a progressive deviation in the balance of the craniocervical junction, which does not allow the head to maintain a neutral position, therefore, impairing the distribution of forces to the TMJ, with potential nasopharyngeal obstruction. The compromised transmission of forces to the temporomandibular articulation leads to dyskinetic masticatory patterns, namely reverse chewing cycles, which further induce malocclusion patterns, exacerbating postural tilt. CCJ: craniocervical junction; TMJ: temporomandibular joint.

Although the complexity of mechanisms involved in the causative relationship between occlusal patterns and postural deficits requires further elucidation, the scientific consensus acknowledges that orthodontic patients often present with an increased Cobb angle, head tilt, or craniocervical inclination, with a significant correlation between malocclusion (specifically with mandible retrusion) and scoliosis (Figure 1) [5,59,62].

Multidisciplinary evaluation and treatment

It is consequential to question whether the correlation between occlusal traits and spinal deformities, such as scoliosis, is strong enough to warrant a multidisciplinary approach when evaluating and treating patients. AIS is characterized by a slow and steady onset of symptoms, some of them discreetly visible during the child’s growth spurt such as uneven hips, chest wall, breasts, shoulders, or the appearance of a shorter lower limb in comparison to the other. Such characteristics can easily go unnoticed especially if the child tends to wear loose-fitting clothing or is not routinely examined by a pediatrician. LBP is not always associated with the early stages of the disease, adding to already existing reasoning for a late diagnosis. Untreated, disease progression includes increasing back pain, balance and mobility impairment, and increasing respiratory restriction [63]. Bracing has been proven to effectively reduce scoliotic curvature and is recommended for curvatures not exceeding 40-45°. Advanced forms of the disease are subject to surgical treatment, and if progressed until adulthood are less likely to be responsive to treatment [64,65]. Early results from a 2011 study examining 52 students with AIS found that 51 of them had varying degrees of malocclusion, while only 19 of them underwent orthodontic treatment [66]. In a case series, Gault demonstrated the benefits of a multidisciplinary evaluation and therapeutic approach, specifically in a 12-year-old female patient diagnosed with mild scoliosis and mandible deviation following orthodontic bracing. Management of this case included the removal of the dental brace and physical therapy for scoliosis, which led to the correct repositioning of the mandible [67].

Interestingly, in the aforementioned study reported by Saccomanno et al., when the scoliotic patients were inquired if their orthopedist or orthodontist informed them about the possibility that scoliosis may influence malocclusion and vice versa, only 12.7% and 11.6%, respectively, replied positively. Within the same cohort, 63.6% of patients confirmed to have had orthodontic therapy before they noticed their scoliosis, 32.5% observed that their teeth became misaligned after scoliosis appeared, and 28.5% stated that their scoliosis appeared during or after orthodontic therapy [59].

Despite the uncertain cause-effect relationship, it could be speculated that orthodontic braces might have effects on the vertebral column and TMJ, impacting postural behavior. In this context, if the scoliosis diagnosis precedes orthodontic treatment, it would appear advisable to delay dental bracing until the end of puberty is reached or to strictly monitor scoliotic curvature development if concomitant treatment is deemed necessary.

## Conclusions

The achievement of perfect occlusion is a complex process requiring multiple factors to align, and the impact of minimal imbalances can have long-lasting effects. The development of orofacial characteristics and TMJ function are linked to head posture as well as spinal alignment, with the association between postural imbalances and malocclusion being scientifically acknowledged. AIS and malocclusion both affect children during their pubertal growth spurt and, while the latter is much easier to diagnose and notice on routine examination, symptoms relating to AIS prove to be discrete and often overlooked. Therapeutic intervention should be undertaken considering attentively the respective development of such coexisting pathologies, as well as the concurrent stage of the patient's growth. Further research must be warranted to provide optimal management and treatment opportunities for patients concomitantly affected by these abnormalities.
